# Organ-Specific Phytochemical Profiling and Antioxidant Analysis of* Parthenium hysterophorus* L.

**DOI:** 10.1155/2018/9535232

**Published:** 2018-06-20

**Authors:** Javed Ahmad, Rita Bagheri, Humayra Bashir, M. Affan Baig, Asma Al-Huqail, Mohamed M. Ibrahim, M. Irfan Qureshi

**Affiliations:** ^1^Department of Biotechnology, Jamia Millia Islamia, New Delhi 110 025, India; ^2^Botany & Microbiology Department, Science College, King Saud University, P.O. Box 2455, Riyadh, Saudi Arabia and Botany & Microbiology Department, Faculty of Science, Alexandria University, P.O. Box 21511, Alexandria, Egypt; ^3^Department of Botany and Microbiology, Faculty of Science, Alexandria University, P.O. Box 51121, Egypt; ^4^Department of Biology and Horticulture, Bergen Community College, Paramus, NJ, USA

## Abstract

*Parthenium hysterophorus *is a weed of global concern with high threshold of tolerance against most of biotic and abiotic stresses. Phytochemical profile and in vitro antioxidant analysis may help in understanding its tolerance to stresses. Root, stem, leaf, phyllary, and receptacle (including disc and ray florets) were chemotyped employing GC tof-MS and assessed for antioxidant activity by DPPH, FRAP, HRSA, and TAC assays. Phytochemicals identified were terpenes, fatty acids, hydrocarbons, phytosterols, and compounds of miscellaneous chemical nature. Organ-specific maximum concentration of metabolite was *β*-vatirenene (root), hexadecanoic acid methylester (stem), aristolene epoxide (leaf), hexadecanoic acid methylester (phyllary), and hexadecanoic acid methylester (receptacle). Identified metabolites could be associated with stress tolerance mechanisms, basic metabolism, and allelopathy, etc. Root extracts showed highest antioxidant potential followed by receptacle. It can be concluded that diverse and unique phytochemical profile and great antioxidant potential make* P. hysterophorus *stress-tolerant, hence a weed of global habitat.

## 1. Introduction

*Parthenium hysterophorus* (Congress grass or Gajar Ghaas, a member of Asteraceae) occur throughout the global agricultural and vacant lands [1] including arid zones. It is an aggressive and invasive weed of cosmopolitan habitat which can tolerate high regimes of stresses including drought and heat [2, 3]. Though* Arabidopsis thaliana*, for most of the studies, has been considered as a model higher plant to study the complex and coordinated molecular basis of abiotic stress tolerance but it may not be considered as a good candidate [4] over some other plants which are much more tolerant against abiotic stresses. There is a special group of plants, ‘weeds', those that invade agriculture fields and compete to important crops through nutrition consumption and allelopathy [5]. Further, weeds are genetically diverse plants which have developed certain degree of tolerance against different stresses.

Our understanding still lags much behind on how weeds genetically and metabolically adapt to abiotic stresses up to such a great threshold, a feature that might not be exhibited by number of plants and thus those are not appropriate models for stress tolerance studies [6]. Therefore,* Parthenium hysterophorus *has been considered as an experimental material in present study.* P. hysterophorus* has special morphophysiological and biochemical adaptabilities and efficient but yet unknown antistress mechanism(s). It has high level of threshold to tolerate biotic and abiotic stresses and capable of biosynthesizing novel secondary metabolites including antioxidants and bioactive allelopathic chemicals for their own defense [7–9]. All these special features enable* P. hysterophorus* to grow well in varied habitats and under harsh and extreme ecological conditions, thus making it a weed of global occurrence (Supplementary Figure S1) [10]. Investigating identity of phytochemicals and antioxidant potential of Parthenium is expected to reveal mechanism of defense and oxyradical quenching [11, 12]. This study was aimed at GC-MS chemotyping and antioxidant activity of different parts of* Parthenium hysterophorus.*

## 2. Materials and Methods

### 2.1. Plant Material

*Parthenium hysterophorus* (Congress grass or Gajar Ghaas) was used as experimental plant. Seeds (Supplementary Figure S2) from authenticated* Parthenium hysterophorus* were obtained from the field of Jamia Millia Islamia (Latitude 28.6° 4'N and Longitude 77.2°), New Delhi, India growing in the alluvial soil. The plant was further identified by Dr. S. K. Srivastava (Scientist E/HOO) of Botanical Survey of India, Dehra Dun, India, where plant specimen has been deposited with Accession no. 115597. Healthy seeds of* Parthenium hysterophorus *were sterilised with 0.3% KMnO_4_ for 10 min and then washed thoroughly with DDW water ten times. Seeds were germinated in dark on moist Soilrite™ and later maintained under 14h/10h light/dark at 25°C with 250 *μ*mol photons m^−2^ s^−1^. Plants (twenty days after germination) were transferred to 6” x 6” size pots filled with Soilrite™ (300 g/pot) with a single plant per pot and further grown for forty days with above-mentioned conditions (Supplementary Figure S3). Thus, two-month-old plants were used in this study (Supplementary Figure S4).

Two-month-old plants were carefully removed from the soil and washed with double distilled water (DDW). Blot dried plants were separated into root, stem, leaf, and flowers. Every organ except flower was immediately immersed in liquid nitrogen. Further, flowers were quickly separated into phyllary and receptacle (including disc and ray florets) with the help of forceps under a flower-dissection microscope that is generally used for taxonomic purpose. Separated phyllary and receptacle were also frozen in liquid nitrogen. The separated organs were then processed for metabolite profiling of* Parthenium hysterophorus *at whole plant level and antioxidant potential of each organ.

### 2.2. Preparation of Metabolite Extracts

Plant organ, namely, root, stem, leaf, phyllary, and receptacle (100 mg each) was pulverised in chilled mortar pestle with liquid nitrogen. The powdered sample was extracted serially in chloroform-acetonitrile-acetone solvents, thrice each at room temperature. The samples were then vacuum-dried to concentrate metabolites. Samples for GC-MS analysis were prepared in acetone, spun at 5000 xg/10 min/4°C, and filtered through 0.45 *μ*m (Millipore, MF) filters before being resolved on GC-MS. Similar extracts were also used for analysis of antioxidant potential. Final concentrations of dried extracts were as follows: control (0.0 *μ*g/ml), 15 *μ*g/ml, 30 *μ*g/ml, 60 *μ*g/ml, and 100 *μ*g/ml.

#### 2.2.1. GC-MS Analytical Conditions

GC-MS instrument (GC-MS-QP2010 Ultra, Shimadzu, Japan) conditions were as follows: auto-sampler: AOC-5000 Plus, LINEX system, column: InertCap Pure-WAX (GL Sciences, Inc., Japan), column oven temperature: 100°C, injection temperature: 270°C, carrier gas: Helium, ion source temperature: 200°C, column flow: 1 mL/min, injection mode: splitless, sampling time: 1.0 min, flow control mode: linear velocity, pressure: 173.1 kPa, total flow: 16.3 mL/min, column flow: 1.21 mL/min, linear velocity: 28.9 cm/sec, purge flow: 3.0 mL/min, split ratio: 10.0, mass range: m/z 40-650, and sample amount: 2.0 *μ*L. Prior running the samples, manual mass calibration was performed using fragment ion m/z 1066 of Tris(perfluorononyl)-S-triazine (molecular weight 1485) as well as mass calibration by autotuning. A mixture of octafluoronaphthalene (1 pg/*μ*L ± 0.01 pg/*μ*L) and benzophenone (10 pg/*μ*L ± 0.1 pg/*μ*L) was run as standard.

#### 2.2.2. GC-MS Data Processing

For GC-MS peak data retrieval, the respective mass spectra of the extracted ion peaks identified by comparison of the total ion current (TIC) mass chromatograms were searched against the NIST (National Institute of Standards and Technology) and Wiley mass spectra libraries (WILLEY8.LIB) and NIST05S.LIB (National Institute of Standards and Technology, Gaithersburg, MD, USA) for similarity matches. This spectral comparison provided putative empirical formulae and structures, which were further searched in databases such as the Dictionary of Natural Products (DNP) (http://dnp.chemnetbase.com) and ChemSpider (http://www.chemspider.com). The identified compounds were catalogued in the form of metabolite library and used for result interpretation. Structures of identified compounds were retrieved from PubChem (NCBI) database. A complete work scheme for phytochemical analysis has been demonstrated in Supplementary Figure S5.

### 2.3. Analysis of Antioxidant Potency of* P. hysterophorus *Organs

#### 2.3.1. 2,2-Diphenyl-1-picryl-hydrazylhydrate (DPPH) Assay

Radical scavenging activity of extracts was measured by the method of Zhou and Yu [13]. Per cent (%) scavenging for each sample was calculated against control (without any sample or extract) as follows:

(1) $$\begin{array}{c} Scavenging\left(\;\%\;\right)=\frac{{OD}_{CONTROL}-{OD}_{SAMPLE}}{{OD}_{CONTROL}}x100 \end{array}$$

#### 2.3.2. Hydroxyl Radical Scavenging Activity (HRSA) Assay

Method of Halliwell et al. [14], with freshly prepared reagents, was used to measure the hydroxyl radical scavenging activity of phytoconstituents. Per cent (%) scavenging for each sample was calculated against control (without any sample or extract) as follows:

(2) $$\begin{array}{c} Scavenging\left(\;\%\;\right)=\frac{OD_{CONTROL}-OD_{SAMPLE}}{OD_{CONTROL}}x100 \end{array}$$

#### 2.3.3. Ferric Reducing Antioxidant Potential (FRAP) Assay

Method of Benzie and Strain [15] was used to study antioxidant potential of* P. hysterophorus *organs extracts. A standard curve (100 – 2000 mol/L) was prepared using FeSO_4_ solution. Values were expressed as *μ*mol Fe^2+^ equivalents (*μ*mol) corresponding to amount of extract per mL.

#### 2.3.4. Total Antioxidant Capacity (TAC) Assay

The total antioxidant capacity of various concentrations of acetonic extracts of* P. hysterophorus *was measured with phosphomolybdenum using ascorbic acid as the standard in 1 mL of TAC reagent as described by Prieto et al. [16]. The values were expressed as ascorbic acid equivalents (*μ*g) corresponding to amount of extract per mL.

### 2.4. Statistical Analysis

For all the experiments three samples were analysed and all the assays were carried out in triplicate. The results were expressed as mean values with standard deviation. Data were analysed using one-way ANOVA and the values of ^*∗*^*P* <0.05 were considered as statistically significant. Besides this, principal component analysis (PCA) and partial least squares discriminant analysis (PLS-DA) were carried out using MetaboAnalyst 3.0 software. Correlation analyses between all metabolite pairs were done using Pearson's correlation.

## 3. Results

Phytochemical profiles of* Parthenium hysterophorus* root, stem, leaves, phyllary, and receptacle were resolved employing Gas Chromatography coupled with Mass Spectrometry (GC-MS) (Supplementary Table 1). GC-MS analyses of the fractionated organs resolved and elevated over 215 peaks in all (Supplementary Figure S6-Figure S10). Approximately, 47% of these peaks were identified (Supplementary Table S2) as metabolites of discrete or common occurrence with known chemical structure (Supplementary Table S3). The identification of the phytochemical compounds was confirmed on the basis of retention time (Rt) and molecular weight (Mr) by submission of signals/peaks to public databases (Wiley and NIST libraries). Metabolites with closest match in different organelles are listed in Supplementary Tables S1 and S2. Results have been presented in five various categories of metabolites along with their derivatives (Supplementary Table S3), namely, terpenes, fatty acids, hydrocarbons, phytosterols, and miscellaneous. These identified metabolites belong to numerous metabolic as well intermediary moieties of specific pathways. A list of most abundant 10 compounds has been provided in Table 1 giving a glimpse of metabolic scenario in different organs.

**Figure 1:**
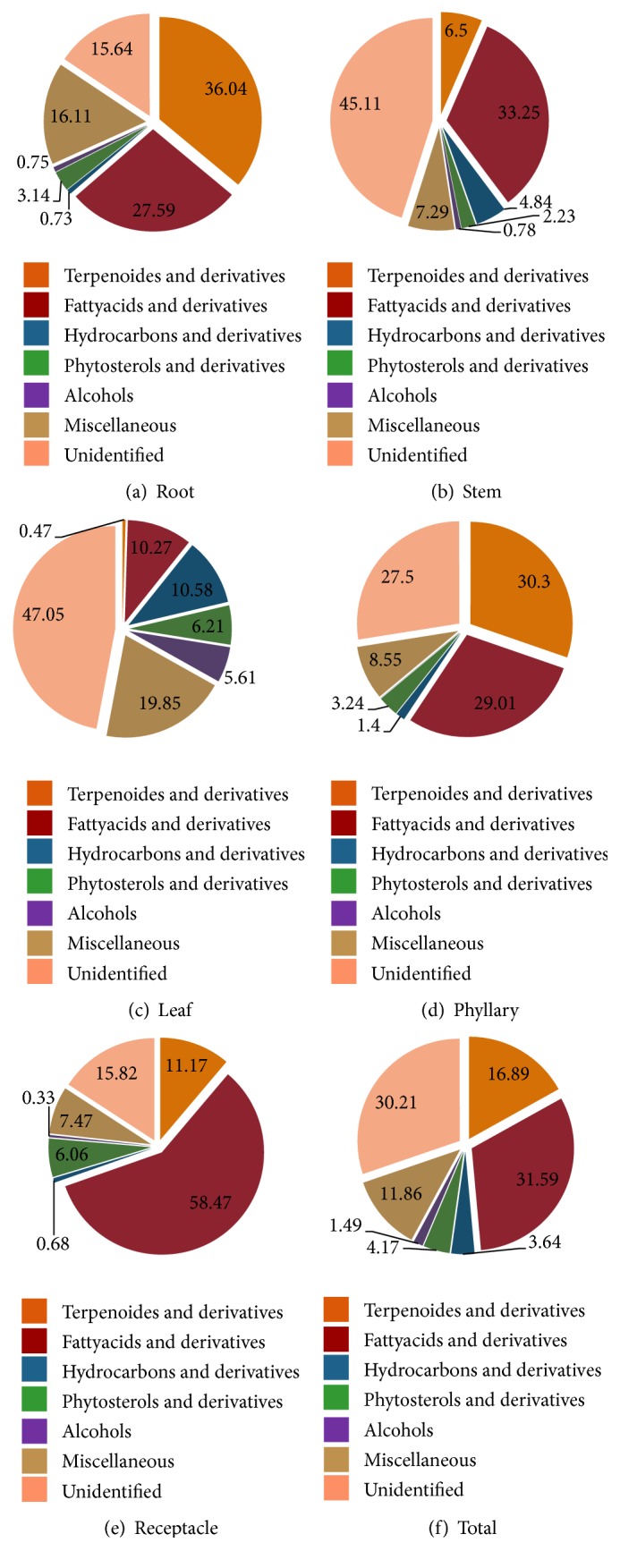
Functional distribution of identified phytocomponents in (a) root, (b) stem, (c) leaf, (d) phyllary, (e) receptacle, and (f) total organs of* Parthenium hysterophorus *expressed in per cent relative metabolite composition per organ.

GC-MS profile based analyses showed the following phytochemicals in different organs (Figures 1(a)–1(f)):** Root:** 25 phytocomponents; 9 terpenoids, 8 fattyacids, 1 hydrocarbons, 1 alcohol, 1 phytosterol, and 5 miscellaneous metabolites (Figure 1(a));** Stem:** 17 phytocomponents; 3 terpenoids, 6 fatty acids, 4 hydrocarbons, 1 alcohol, 1 phytosterol, and 2 miscellaneous metabolites (Figure 1(b));** Leaf:** 47 phytocomponents; 3 terpenoids, 14 fatty acids, 4 hydrocarbons, 7 alcohols, 5 phytosterols, and 14 other metabolites (Figure 1(c));** Phyllary:** 31 phytocomponents; 11 terpenoids, 12 fatty acids, 1 hydrocarbons, 2 phytosterols, and 5 others miscellaneous metabolites (Figure 1(d));** Receptacle:** 24 phytocomponents; 6 terpenoids, 9 fatty acids, 1 hydrocarbons, 1 alcohol, 3 phytosterol, and 4 miscellaneous metabolites (Figure 1(e)). Overall, 17% terpenes/derivatives, 32% fatty acids/derivatives, 4% hydrocarbons/derivatives, 1.5% alcohols, 4% phytosterol/derivatives, and 12% miscellaneous metabolites could be identified in* P. hysterophorus* (Figure 1(f)).

### 3.1. Antioxidant Analysis of* P. hysterophorus *Organs

**Figure 2:**
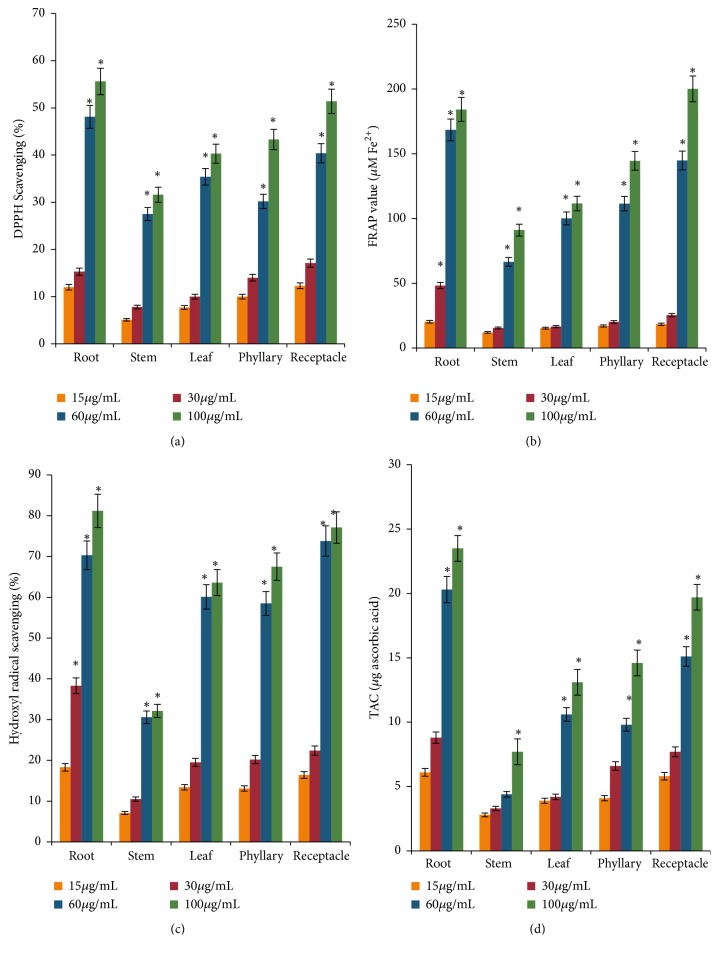
Impact of* Parthenium hysterophorus* root, stem, leaf, phyllary, and receptacle acetonic extracts on (a) DPPH^.^ scavenging, (b) ferric reducing antioxidant activity, (c) hydroxyl radical scavenging, and (d) total antioxidant activity. Values are expressed as mean ± standard deviation (n = 3, ^*∗*^P<0.05).

Different extracts of* P. hysterophorus* showed a concentration-dependent scavenging potential, maximum by root and least by stem (Figure 2(a)).

Ferric reducing antioxidant activity of different* P. hysterophorus* extracts increased in a concentration-dependent manner, maximum by root/ receptacle and least by stem (Figure 2(b)).

Different organs of* P. hysterophorus* exhibited varied scavenging potential of OH· free radicals but in concentration-dependent manner, maximum by root/ receptacle and least by stem (Figure 2(c)).

The total antioxidant activity of the extract was determined in terms of ascorbic acid equivalence. Results suggested that root and stem exhibited highest and least antioxidant activity, respectively (Figure 2(d)).

### 3.2. Multivariate Data Analysis

**Figure 3:**
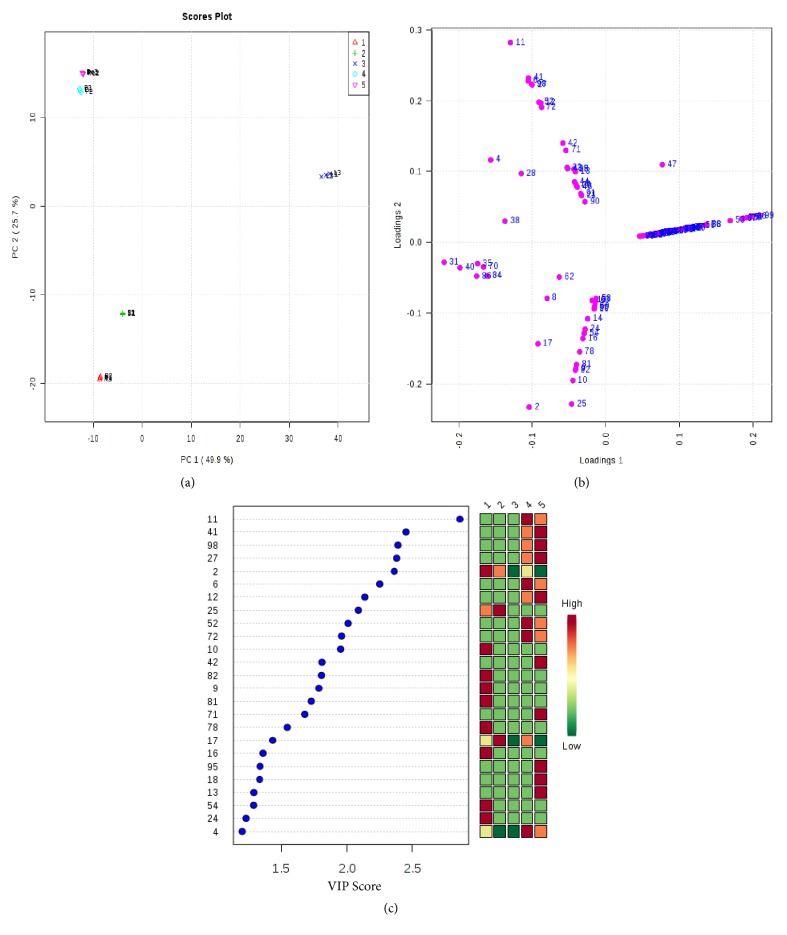
Multivariate analysis of the data using MetaboAnalyst software. Scores plot (a) and loadings plot (b) of PCA analysis showing correlation between different metabolites found in different organs. Variable importance in projection (VIP) plot (c) demonstrating metabolites with maximum abundance in different organs: 1= root; 2 = stem; 3 = leaf; 4 = phyllary; 5 = receptacle.

**Figure 4:**
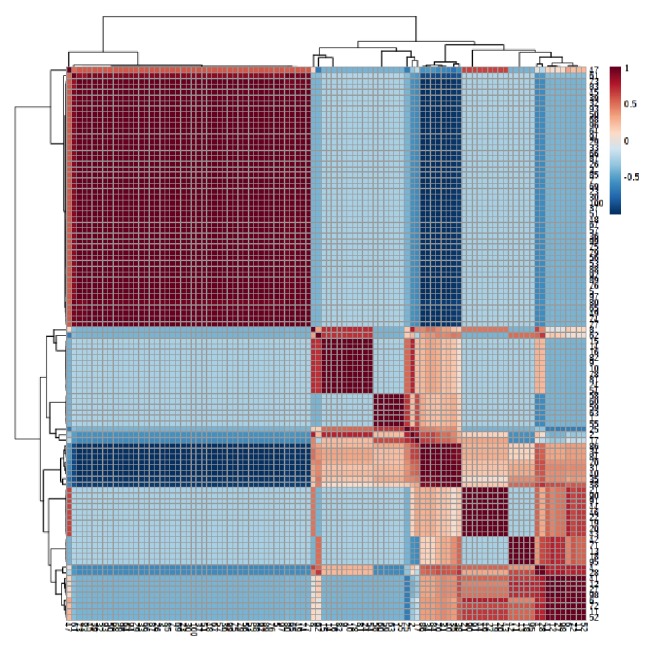
Hierarchical cluster investigation. Heat map analysis of the metabolite-metabolite correlation found in different organs of* P. hysterophorus *using Pearson's correlation coefficient.

For normalizing the scale of abundance, percentage volumes of each metabolite were log-transformed to base 2 prior to data analysis using MetaboAnalyst software. The unsupervised PCA loadings and scores plots allowed the visualization of data and parallel comparison of the differentially modulated metabolites among different organs (Figures 3(a) and 3(b)). PCA plots showed a clear separation of metabolites in different PCs, further confirming the differential modulation of these selected metabolites (Figure 3(a)). To identify the metabolites showing maximum abundance change among the root, stem, leaf, phyllary, and receptacle stages, a VIP-plot was constructed from the loading plots of PLS-DA (Figure 3(c)). VIP-plots identified a total of 25 metabolites with VIP scores (1.1–2.9) (Figure 3(c)). Different metabolites were higher in different organs (marked with red color in Figure 3(c)), suggesting their pivotal role in respective organs. The scores plot of the PCA analysis showed clear groupings of the different organs. Variation in the dataset in different organs can be explained by principal component 1 (PC1) which accounts for 49.9% of the variation. These results showed that organ-specific metabolite changes. Correlation analyses were carried out to identify the relationship among the metabolites detected in the different organs of* P. hysterophorus*. The heatmaps of the correlation analysis provide an overview of the correlation in each tissue for different metabolites (Figure 4(a)). In all organs, most of the metabolites measured showed positive correlation to each other. Metabolites belong to terpenoids (Steviol, Caryophyllene oxide, Spathulenol, Globulol, Longifolenaldehyde, Bergamotol, Lanceol, and Chrysanthemol) were consistently positively correlated with the majority of the phytosterols (Stigmasterol, *β*-Sitosterol, Brassicasterin, and Gamma-Sitosterol) in all organs. These groups of metabolites could be of great significance in nonfriendly environments for adaptation and defense of plants.

## 4. Discussion

Gas chromatography-mass spectrometry (GC-MS) is a quality tool for metabolites analysis since it provides a comprehensive, unbiased, and nearly accurate identification of most metabolites present in a biological sample. Information obtained would help in elucidation of organelle physiochemical status and associated molecular features, being identified as metabolic biomarkers for growth stage and environment responses [17]. Another important aspect is differential distribution patterns of secondary metabolites which indicate about each class of compounds playing roles in individual organs, as revealed in the present study on* Parthenium hysterophorus*, member of Asteraceae (a plant family with enormous number of identified active compounds on public database). In different organs, namely, root, stem, leaf, phyllary, and receptacle, 100 phytochemicals of diverse chemical nature were identified (Supplementary Table 1 and Table 2). Hierarchical clustering analysis (HCA) (Supplementary Figure S11) further provided relations within the group of phytochemicals.

Among identified phytochemicals was the presence of high concentration of *β*-vatirenene (19.8% in root). In a very recent report Koo* et al.* [18] have shown the presence of this metabolite in breath tests of patients suffering from aspergillosis. We suspect and suggest that the study region of Koo et al. [18] inhabits* P. hysterophorus *which secretes *β*-vatirenene into the soils. It is quite possible that *β*-vatirenene reaches to the lungs through dust and makes them prone for aspergillosis; however, it needs a validation. Caryophyllene oxide is shown as a potent antifungal agent [19] providing resistance to reproductive organs and root against fungal infections as also mentioned by Zaheer et al. [20]. Spathulenol (present in phyllary and receptacle) can also be attributed with similar functions [21]. Globulol exhibits potent antibacterial activity [22]. Presence of andrographolide (6.6% in root) may be attributed to growth inhibition of plants growing near* P. *hysterophorus perhaps by inhibiting transcriptional activity, cell cycle arrest, and causing apoptosis [23]. Longifolenaldehyde [24], an oily liquid, seems responsible for resistance to flowers against desiccation Squalene, found mainly in stem, and is a highly unsaturated hydrocarbon from triterpenoid family which not only works as a moisturiser but is also antipathogenic [25]. Presence of methyl erucate, olealdehyde, methy lignocerate, and epicedrol, eicosanoic acid, arachidonic acid, behenic acid, linolenic acid, and palmitic acid help plant adapt to stress [26]. Behenic acid is long-chain saturated fatty acid serving a basic and fluidic function in organs. Linolenic acid methy ester content was high in most organs suggesting dependence on this basic but important fatty acid.

Myristylaldehyde (tetradecanal) and related phytochemicals occur in waxy cuticle present on aerial surfaces of higher plants [27, 28]. 1-Heneicosyl formate was present in receptacle which has been reported from leaf, wood, and floral parts of various plants [29]. Eicosyne scavenges free radicals. 1-Pentadecene is a part of essential oil [30] and shown to accumulate under stress. 1-Nonadecene, app. 7% in leaf, is expected to protect leaf from heat stress as observed in a bacteria by 2-fold accumulation [31]. Hexatriacontane, a plant wax derived alkane, perhaps gives protection to stem and heneicosane which bears antimicrobial activities [32] that might save stem from infections.

Aristolene epoxide, present only in leaf, is a volatile compound of essential oils. The phytochemical 3,7,11,15-tetramethyl-2-hexadecen-1-ol (phytol) is a unsaturated long-chain fatty acid alcohol which was found in leaf and phyllary. This phytochemical is known for antimicrobial activity [33] and might be contributing in protection against microbes with a significant protection to phyllary. Stigmasterol which occurred between 2 and 3% in all organs except leaf indicated its lesser susceptibility to bacterial infection [34]. 2,6-Di-*tert*-butylphenol is a precursor to more complex compounds used as antioxidants and light-protection agents. Brassicasterol is synthesised by marine algae (e.g., diatoms) and some plants. It has got affinity to water and hence could be potentially involve in stem water retention. However, such sterols when administered by human cross every barrier to reach brain and could be associated with Alzheimer's disease [35]. Dibutyl phthalate and Di-n-octyl phthalate seem to provide defense to almost every organ [36].

In addition to phytochemical profiling, present study also evaluated the antioxidant potential of different parts of* P. hysterophorus*. Based on GC tof-MS metabolic profiling results, it can be concluded that* P. hysterophorus* has phytochemicals of diverse nature which might help in exhibiting a variation in antioxidant activities. Our findings show similarity with the previous observations of Pandey and Kumar [37] that plants possessing bioactive compounds of diverse nature have strong antioxidant activity. The DPPH assay determines the scavenging of stable radical species DPPH by antioxidant compounds present in the extracts. The results showed the greater rate of DPPH scavenging activity by root followed by receptacle, leaf, and stem extracts was in the same pattern as in hydroxyl free radical scavenging. This is probably due to the presence of variable range of terpenes, fatty acids, sterols, and their derivatives in different parts of Parthenium. Here terpenes and terpenoids emerge as a major antioxidant combating oxidative stress by donating hydrogen to free radicals. FRAP assay represented direct correlation between high reducing power and high content of phytoconstituents in receptacle and root extract. Similar findings of FRAP assay in root extract were shown by Thusoo et al. [38] which strongly supported our results. The more appreciable TAC value was observed in root and receptacle extracts might be contributed primarily by beta-vatirenene, andrographolide, hexadecanoic acid methyl ester, stigmasterol caryophyllene oxide, longifolenaldehyde, epicedrol, and *β*-sitosterol which represented the significance of secondary metabolites in* Parthenium hysterophorus*.

## 5. Conclusion

Metabolic profile of different organs of* Parthenium hysterophorus *exhibits the occurrence of phytochemicals associated with number of functions including normal growth, development, and defense. A high content of *β*-vatirenene (app. 20% relative content) in root is supposed to be an allergen. Lots of oily and waxy compounds identified in stem, leaf, phyllary, and receptacle make a perfect blend to equip them with glaze on surfaces, softness, flexibility, and water retention to improve the ‘weediness' characters of* P. hysterophorus*. Different parts of* P. hysterophorus* possess considerable amount of in vitro antioxidant activity. The antioxidant activities were almost proportional to the number and amount of different phytochemicals present in different organs rather than quantity of any single compound representing protective effect against oxidative damage. These altogether provide a great degree of antistress mechanism and antioxidant-mediated self-defense that makes this plant a leader among invasive weeds and a perfect model for studying stress tolerance mechanisms.

## Figures and Tables

**Table 1:** Top ten most abundant compounds in different organs of *Parthenium hysterophorus*. Parenthesis include per cent (%) of total concentration.

**S.No.**	**Root** **(%)**	**Stem** **(%)**	**Leaf** **(%)**	**Phyllary** **(%)**	**Receptacle** **(%)**
1.	Beta-vatirenene (19.84)	Hexadecanoic acid, methyl ester (15.68)	Aristolene epoxide (10.41)	Hexadecanoic acid, methyl ester (12.04)	Hexadecanoic acid, methyl ester (23.04)

2.	Hexadecanoic acid, methyl ester (12.05)	Linolenic acid methyl ester (8.18)	Hexatriacontane (7.47)	Longifolenaldehyde (11.55)	Linolenic acid methyl ester (10.11)

3.	Linolenic acid methyl ester (7.32)	Di-n-octyl phthalate (5.18)	1-Nonadecene(6.75)	Caryophyllene oxide (7.89)	Stearic acid, methyl ester (8.62)

4.	Andrographolide (6.65)	Stearic acid, methyl ester (4.81)	2,5-Di-tert-butylphenol (5.90)	Linolenic acid methyl ester (5.53)	Longifolenaldehyde (4.06)

5.	*β*-D-Lyxofuranoside, 5-O-(beta.-D-lyxofuranosyl)-decyl (4.09)	Squalene (4.00)	Brassicasterin (5.08)	Di-n-octyl phthalate (4.43)	1-Dimethyl-isopropylsilyloxynonane (3.08)

6.	Di-n-octyl phthalate (3.90)	Stigmasterol (2.23)	3,7,11,15-Tetramethyl-2-hexadecen-1-ol (4.75)	1,2-Benzenedicarboxylic acid, butyl 2-ethylhexyl ester (2.78)	Olealdehyde(3.07)

7.	Globulol (3.83)	Dibutyl phthalate (2.11)	1-Pentadecene (2.80)	Stigmasterol (2.28)	Behenic acid methyl ester (3.01)

8.	1,2-Benzenedicarboxylic acid, butyl 2-ethylhexyl ester (3.24)	Heneicosane (1.47)	Gamma.-Sitosterol (0.92)	*α*-Amyrin acetate (1.84)	Myristylaldehyde (3.00)

9.	Dibutyl phthalate (3.24)	Tetratriacontane (1.43)	1,4-Eicosadiene (0.72)	m-Anisic acid, tridec-2-ynyl ester (1.70)	Caryophyllene oxide(2.78)

10.	Stigmasterol (3.14)	Hexatriacontane (1.26)	Triacontanol (0.56)	Eicosyne (1.40)	Stigmasterol(2.68)

## Data Availability

The data used to support the findings of this study are available from the corresponding author upon request.

## Supplementary Materials

Supplementary Materials Figure S1: schematic representation of* Parthenium hysterophorus* distribution pattern in various geographical regions of the world. Figure S2: seeds of* Parthenium hysterophorus* (black colored) collected from field of Jamia Millia Islamia, New Delhi, India. Figure S3: different growth stages during mass cultivation of* Parthenium hysterophorus* under controlled growth conditions in soil (Soilrite™) culture which was fed according to WHC with half strength of Hoagland nutrient media. Figure S4:* Parthenium hysterophorus* plants at the age of sixty days which is a flowering stage and used for phytochemical analysis. Figure S5: a schematic representation of work plan adopted for metabolomic profiling of different parts of* Parthenium hysterophorus. *For phytochemical profiling, flowers were harvested at plant age of two months. Plants were divided into phyllary and receptacle. Thus, phytochemical profiling was done for root, stem, leaf, phyllary, and receptacle. Supplementary Table S1: different phytochemicals identified by GC-MS in different organs of* Parthenium hysterophorus* mentioned with their relative content. Parenthesis includes per cent of total metabolite amount identified in the organ. Supplementary Table 2: list of phytocomponents along with their corresponding RT, molecular formula, molecular weight, and structure identified in different organelles of* Parthenium hysterophorus *by GC-MS. Supplementary Table 3: nature of the phytocompounds identified in the acetonic extracts of* Parthenium hysterophorus* by GC-MS. Figure S6: the GC-MS chromatogram of acetonic extract of* Parthenium hysterophorus* roots showing the peaks of the test compounds versus retention time in minutes. Figure S7: the GC-MS chromatogram of acetonic extract of* Parthenium hysterophorus* stem showing the peaks of the test compounds versus retention time in minutes. Figure S8: the GC-MS chromatogram of acetonic extract of* Parthenium hysterophorus* leaf showing the peaks of the test compounds versus retention time in minutes. Figure S9: the GC-MS chromatogram of acetonic extract of* Parthenium hysterophorus* phyllary showing the peaks of the test compounds versus retention time in minutes. Figure S10: the GC-MS chromatogram of acetonic extract of* Parthenium hysterophorus* receptacle showing the peaks of the test compounds versus retention time in minutes. Figure S11: hierarchical clustering analysis (HCA) of metabolites identified in five different organs of* Parthenium hysterophorus* by Log2-transformed and* Z*-scored signal intensity data were hierarchically classified using the average linkage clustering methods. Metabolites not detected in any of the organs are indicated in gray. High and low spot abundance ratios. Metabolite names are shown on the right side. Red color indicates high abundance, whereas low relative metabolites are dark brown. Black boxes indicate metabolite clusters with similar abundance. 1 = root, 2 = stem, 3 = leaf, 4 = phyllary, and 5 = receptacle.
