# Supplementary material for: Organ-Specific Phytochemical Profiling and Antioxidant Analysis of Parthenium hysterophorus L
Source: Biomed Res Int. 2018 Jun 20;2018:9535232. doi: 10.1155/2018/9535232 (PMC6031210; doi:10.1155/2018/9535232)
Supplement: Supplementary Materials — Figure S1: schematic representation of Parthenium hysterophorus distribution pattern in various geographical regions of the world. Figure S2: seeds of Parthenium hysterophorus (black colored) collected from field of Jamia Millia Islamia, New Delhi, India. Figure S3: different growth stages during mass cultivation of Parthenium hysterophorus under controlled growth conditions in soil (Soilrite™) culture which was fed according to WHC with half strength of Hoagland nutrient media. Figure S4: Parthenium hysterophorus plants at the age of sixty days which is a flowering stage and used for phytochemical analysis. Figure S5: a schematic representation of work plan adopted for metabolomic profiling of different parts of Parthenium hysterophorus. For phytochemical profiling, flowers were harvested at plant age of two months. Plants were divided into phyllary and receptacle. Thus, phytochemical profiling was done for root, stem, leaf, phyllary, and receptacle. Supplementary Table S1: different phytochemicals identified by GC-MS in different organs of Parthenium hysterophorus mentioned with their relative content. Parenthesis includes per cent of total metabolite amount identified in the organ. Supplementary Table 2: list of phytocomponents along with their corresponding RT, molecular formula, molecular weight, and structure identified in different organelles of Parthenium hysterophorus by GC-MS. Supplementary Table 3: nature of the phytocompounds identified in the acetonic extracts of Parthenium hysterophorus by GC-MS. Figure S6: the GC-MS chromatogram of acetonic extract of Parthenium hysterophorus roots showing the peaks of the test compounds versus retention time in minutes. Figure S7: the GC-MS chromatogram of acetonic extract of Parthenium hysterophorus stem showing the peaks of the test compounds versus retention time in minutes. Figure S8: the GC-MS chromatogram of acetonic extract of Parthenium hysterophorus leaf showing the peaks of the test compounds versus retenti [file 9535232.f1.docx]

Figure S1. Schematic representation of *Parthenium hysterophorus* distribution pattern in various geographical regions of the World.

Figure S2. Seeds of *Parthenium hysterophorus* (black coloured) collected from field of Jamia Millia Islamia, New Delhi, India.

Figure S3. Different growth stages during mass cultivation of *Parthenium hysterophorus* under controlled growth conditions in soil (Soilrite™) culture which was fed according to WHC with half strength of Hoagland nutrient media.

Figure S4. *Parthenium hysterophorus* plants at the age of sixty days which is a flowering stage and used for phytochemical analysis.

Figure S5. A schematic representation of work plan adopted for metabolomic profiling of different parts of *Parthenium hysterophorus.* For phytochemical profiling, flowers were harvested at plant age of two months. Plants were divided into phyllary and receptacle. Thus, phytochemical profiling was done for root, stem, leaf, phyllary and receptacle.

Supplementary Table S1. Different phytochemicals identified by GC-MS in different organs of *Parthenium* *hysterophorus* mentioned with their relative content. Parenthesis includes per cent of total metabolite amount identified in the organ.

Supplementary Table 2. List of phytocomponents along with their corresponding RT, molecular formula, molecular weight and structure identified in different organelles of *Parthenium hysterophorus* by GC-MS.

Supplementary Table 3. Nature of the phytocompounds identified in the acetonic extracts of *Parthenium hysterophorus* by GC-MS*.*

Figure S6. The GC-MS chromatogram of acetonic extract of *Parthenium hysterophorus* roots showing the peaks of the test compounds Vs retention time in minutes.

Figure S7. The GC-MS chromatogram of acetonic extract of *Parthenium hysterophorus* stem showing the peaks of the test compounds Vs retention time in minutes.

Figure S8. The GC-MS Chromatogram of acetonic extract of *Parthenium hysterophorus* leaf showing the peaks of the test compounds Vs retention time in minutes.

Figure S9. The GC-MS Chromatogram of acetonic extract of *Parthenium hysterophorus* phyllary showing the peaks of the test compounds Vs retention time in minutes.

Figure S10. The GC-MS Chromatogram of acetonic extract of *Parthenium hysterophorus* receptacle showing the peaks of the test compounds Vs retention time in minutes.

Figure S11: Hierarchical clustering analysis (HCA) of metabolites identified in five different organs of *Parthenium hysterophorus* by Log2-transformed and *Z*-scored signal intensity data were hierarchically classified using the average linkage clustering methods. Metabolites not detected in any of the organs are indicated in gray. High and low spot abundance ratios. Metabolite name are shown on the right side. Red colour indicates high abundance, whereas low relative metabolites are dark brown. Black boxes indicate metabolite clusters with similar abundance. 1 = root, 2 = stem, 3 = leaf, 4 = phyllary and 5 = receptacle.
